# Identifying Complex lncRNA/Pseudogene–miRNA–mRNA Crosstalk in Hormone-Dependent Cancers

**DOI:** 10.3390/biology10101014

**Published:** 2021-10-09

**Authors:** Dulari K. Jayarathna, Miguel E. Rentería, Emilie Sauret, Jyotsna Batra, Neha S. Gandhi

**Affiliations:** 1 Centre for Genomics and Personalised Health, School of Chemistry and Physics, Queensland University of Technology, Brisbane, QLD 4000, Australia; dulari.lekamlage@hdr.qut.edu.au (D.K.J.); jyotsna.batra@qut.edu.au (J.B.); 2 Department of Genetics and Computational Biology, QIMR Berghofer Medical Research Institute, Brisbane, QLD 4006, Australia; miguel.renteria@qimrberghofer.edu.au; 3 School of Biomedical Sciences, Queensland University of Technology, Brisbane, QLD 4059, Australia; 4 School of Mechanical, Medical & Process Engineering, Queensland University of Technology, Brisbane, QLD 4000, Australia; emilie.sauret@qut.edu.au; 5 Translational Research Institute, Brisbane, QLD 4102, Australia

**Keywords:** hormone-dependent cancers, ceRNAs, lncRNAs, microRNAs, pseudogenes, multiple sensitivity correlation

## Abstract

**Simple Summary:**

Competing endogenous RNAs (ceRNAs) have gained attention in cancer research owing to their involvement in microRNA-mediated gene regulation. Here, we identified a shared ceRNA network across five hormone-dependent (HD) cancers (prostate, breast, colon, rectal, and endometrial), that contain two long non-coding RNAs, nine mRNAs, and seventy-four microRNAs. Among them, two mRNAs and forty-one microRNAs were associated with at least one HD cancer survival. A similar analytical approach can be applied to identify shared ceRNAs across a group of related cancers, which will significantly contribute to understanding their shared disease biology.

**Abstract:**

The discovery of microRNAs (miRNAs) has fundamentally transformed our understanding of gene regulation. The competing endogenous RNA (ceRNA) hypothesis postulates that messenger RNAs and other RNA transcripts, such as long non-coding RNAs and pseudogenes, can act as natural miRNA sponges. These RNAs influence each other’s expression levels by competing for the same pool of miRNAs through miRNA response elements on their target transcripts, thereby modulating gene expression and protein activity. In recent years, these ceRNA regulatory networks have gained considerable attention in cancer research. Several studies have identified cancer-specific ceRNA networks. Nevertheless, prior bioinformatic analyses have focused on long-non-coding RNA-associated ceRNA networks. Here, we identify an extended ceRNA network (including both long non-coding RNAs and pseudogenes) shared across a group of five hormone-dependent (HD) cancers, i.e., prostate, breast, colon, rectal, and endometrial cancers, using data from The Cancer Genome Atlas (TCGA). We performed a functional enrichment analysis for differentially expressed genes in the shared ceRNA network of HD cancers, followed by a survival analysis to determine their prognostic ability. We identified two long non-coding RNAs, nine genes, and seventy-four miRNAs in the shared ceRNA network across five HD cancers. Among them, two genes and forty-one miRNAs were associated with at least one HD cancer survival. This study is the first to investigate pseudogene-associated ceRNAs across a group of related cancers and highlights the value of this approach to understanding the shared molecular pathogenesis in a group of related diseases.

## 1. Introduction

MicroRNAs (miRNAs) are endogenous non-coding RNAs consisting of 19–25 nucleotides in length. They regulate gene expression through the degradation or inhibition of translation by binding to messenger RNA (mRNA) [1]. A single miRNA can target hundreds of genes. Therefore, miRNAs play a crucial post-transcriptional role in DNA–RNA–protein networks. In each cell, transcripts such as mRNAs, long non-coding RNAs (lncRNAs), and pseudogenes contain similar miRNA response elements (MREs) that can crosstalk via competition of binding to common miRNAs serving as miRNA sponges. In 2011, this phenomenon was described as “the competing endogenous RNA (ceRNA) hypothesis” [2]. As a major ceRNA component, lncRNA has a dual role in the nucleus and cytoplasm. Several studies suggest that lncRNAs directly interact with transcription factors as transcriptional co-activators in the nucleus, while others suggest that lncRNAs may impair transcriptional complexes’ assembly as an inhibitor of gene expression [3]. The pseudogenes are very similar to the coding genes, as they are produced by modifying and cutting off the coding transcripts in the transcription process. Both pseudogenes and cytoplasmic lncRNAs (not in the nucleus) act as regulators to affect their target genes [4]. These ceRNAs, lncRNAs, and pseudogenes may influence cancer pathogenesis by regulating mRNA expression of crucial tumorigenic or tumour-suppressive genes and pathways [5].

Previous bioinformatics studies have identified ceRNA candidates as prognostic or predictive biomarkers for common cancer types such as colorectal, endometrial, prostate, and breast cancers [6,7,8,9,10]. Several web-based tools such as miRTissue_ce_, LncACTdb 2.0, and lnCeDB have been developed, supporting the search for ceRNA interaction networks in multiple tissues [11,12,13]. A recent colorectal cancer ceRNA study identified a network of nine hub genes, thirteen lncRNAs, and twenty-nine candidate miRNAs, integrating multiple genomic datasets [6]. The authors further revealed the *MFAP5*-miR-200b-3p-AC005154.6 axis as a potential biomarker of colorectal cancer. In 2019, bioinformatic analyses conducted by Wang et al. [7] and Ouyang et al. [8] revealed two endometrial-cancer-associated ceRNAs, lncRNA LINC00958 (*DOLPP1*-miR-761-LINC00958) and lncRNA LINC00261 (*C2orf48*-LINC00261), respectively. Recent experimental studies have validated that these two lncRNAs act as critical regulators of endometrial cancer, binding through multiple mRNA–miRNA axes [14,15]. A ceRNA network analysis of prostate cancer established a network consisting of four hub genes, homeobox B5 (*HOXB5*), glypican 2 (*GPC2*), pepsinogen A-5 (*PGA5*), and ameloblastin (*AMBN*), which are strongly associated with patient survival [9]. A comprehensive lncRNA-associated ceRNA analysis of breast cancer identified ninety-three lncRNAs, twenty-seven mRNAs, and nineteen miRNAs. In this dataset, fifteen lncRNAs were identified as prognostic biomarkers of breast cancer [10]. The studies described above suggest that existing ceRNA network analyses can be successfully applied to distinct cancer types in order to understand their biological mechanisms further. Identifying common ceRNA networks across genetically related diseases such as hormone-dependent (HD) cancers will also significantly contribute to understanding the shared molecular pathogenesis.

This study identifies a shared ceRNA network across HD cancers, including prostate, breast, colon, rectal, and endometrial, which are among the world’s highest cancer mortality and incident rates.

## 2. Materials and Methods

### 2.1. Ethics Statement

The Human Research Ethics committees of the Queensland University of Technology (protocol code: 1900001147, date of approval: 19 December 2019) and the QIMR Berghofer Medical Research Institute (protocol code: P1051, date of approval: 23 August 2019) approved this study.

### 2.2. Patients and Samples

RNA expression data (RNA-seq and miRNA-seq) and clinical data for five HD cancers, prostate (PRAD), breast (BRCA), colon (COAD), rectal (READ), and endometrial (UCEC), were obtained from The Cancer Genome Atlas (TCGA). The PRAD, BRCA, COAD, READ, and UCEC consist of 499/52 (cases/controls), 1109/113, 480/41, 167/10, and 552/35, respectively. The HTSeq-count RNA-seq data and isoform quantification data of miRNA-seq for the given five cancer types were downloaded to a local computing server from the GDC (Genomics Data Commons) data portal [16].

### 2.3. Differential Expression Analysis of Hormone-Dependent Cancer Data

**Figure 1:**
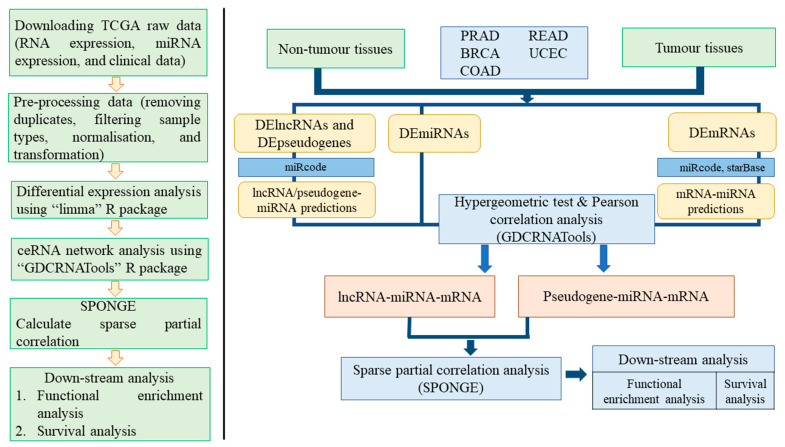
Methodological workflow of the study. RNA-seq and miRNA-seq data extracted from The Cancer Genome Atlas (TCGA) were pre-processed, normalised, and transformed into a standard scale. Differential expression analysis was conducted to identify differentially expressed (DE) long non-coding RNAs (lncRNAs), pseudogenes, messenger RNAs (mRNAs), and microRNAs (miRNAs). The competing endogenous RNA (ceRNA) network analysis followed three steps: (i) identifying lncRNA/pseudogene–mRNA pairs sharing the significant number of miRNAs, (ii) calculating the Pearson correlation between lncRNA/pseudogene and mRNAs, and (iii) calculating multiple sensitivity correlation considering a set of miRNAs targeted by a given lncRNA/pseudogene–mRNA pair. The first two steps were conducted using the GDCRNATools R/Bioconductor package [19]. For step iii, sparse partial correlation analysis was executed using the SPONGE (sparse partial correlation on gene expression) R/Bioconductor package [20]. The three-step analysis filtered out statistically significant ceRNAs for individual HD cancers. Then, only shared ceRNA components across all five HD cancers were involved in the downstream analysis.

At the data pre-processing stage, we removed TCGA samples with duplicated sample IDs. Then, metastatic samples were eliminated as we compared primary tumours and adjacent normal samples using differential expression analysis. The raw count expression data were normalised by the TMM (trimmed mean of M values) method implemented in the edgeR R package [17]. The normalised data were transformed into a standard scale using the voom method implemented in the limma (linear modelling for microarrays) R package [18]. Low-expressed genes (log counts per million < 1 in more than 50% of the samples) were removed by default. Ignoring low-expressed genes increases the total count of differentially expressed genes after multiple testing correction and improves sensitivity and precision. Genes or miRNAs that were differentially expressed between tumours and adjacent normal tissues were identified by applying “lmFit” followed by “eBayes (empirical Bayes)”, in-built functions in the limma R package [18]. We fitted a linear model for each gene using the “lmFit” function. Then, eBayes moderation was applied, borrowing information across all the genes to obtain more precise estimates of gene-wise variability. Expression differences were assessed by linear modelling results: log fold-change (logFC) and false discovery rate (FDR)-adjusted *p*-values. |logFC| > 1 and FDR < 0.01 were considered thresholds with which to identify statistically significant mRNAs, lncRNAs, pseudogenes, and miRNAs. Differentially expressed lncRNAs, pseudogenes, and mRNAs were separately recorded for ceRNA network analysis. Figure 1 depicts the workflow of this study.

### 2.4. Competing Endogenous RNA Network Analysis

Initially, we constructed both lncRNA-based and pseudogene-based ceRNA networks for individual HD cancers. Then, we identified shared ceRNA associations across five HD cancers. Two downstream analyses, functional enrichment and survival analyses, were conducted for shared genes, lncRNAs, pseudogenes, and miRNAs across HD cancers ceRNA networks.

### 2.5. Long Non-Coding RNA/Pseudogene–mRNA–microRNA Networks

We followed three steps to identify ceRNA interactions, (i) detecting lncRNA/pseudogene–mRNA pairs that share a significant number of miRNAs, (ii) selecting positively correlated lncRNA/pseudogene–mRNA pairs, and (iii) jointly estimating the significance of multiple miRNAs in lncRNA/pseudogene–mRNA pairs. The miRNA–mRNA, miRNA–lncRNA, and miRNA–pseudogene interactions required for steps i and iii were obtained from two databases, miRcode and starBase [21,22]. The miRcode database facilitates mRNA–miRNA, lncRNA–miRNA and pseudogene–miRNA target predictions using a broad searchable map that contains 10,419 lncRNAs and 12,549 pseudogenes. The starBase includes miRNA–mRNA interactions predicted by probing 108 CLIP-seq datasets. As described above, a similar three-step approach has been previously followed by the miRTissue_ce_, a ceRNA–ceRNA web application tool [11]. In the first step, we used a hypergeometric test to identify lncRNA/pseudogene–mRNA pairs with a significant number of shared miRNAs. The hypergeometric-test-associated *p*-value can be computed using the following equation, Equation (1):

(1) $$p=1-\overset{m}{\underset{k=0}{\sum }}\frac{\left(\begin{array}{c} K\\ k \end{array}\right)\left(\begin{array}{c} N-K\\ n-k \end{array}\right)}{\left(\begin{array}{c} N\\ n \end{array}\right)}\;$$

where *m* is the number of shared miRNAs, *N* is the total number of available miRNAs, *n* is the number of miRNAs targeting the lncRNA/pseudogene, and *K* is the number of miRNAs targeting the mRNA. MiRNAs are known as negative regulators of gene expression. If an lncRNA/pseudogene occupies the majority of miRNAs, only a small proportion is available to bind to the target mRNA, increasing the mRNA’s expression level. Based on this phenomenon, the lncRNA/pseudogene–mRNA pair should be positively correlated. As the second step, we applied the Pearson correlation analysis to extract positively correlated lncRNA/pseudogene–mRNA pairs from all possible lncRNA/pseudogene–mRNA interactions. Both the hypergeometric test and Pearson correlation analysis were carried out using the GDCRNATools R/Bioconductor package [19]. In GDCRNATools, the regulation contribution towards a ceRNA interaction has been quantified using the sensitivity correlation (scor) [23]. The scor value does not account for a combinatorial effect of multiple miRNAs. Subsequently, strong ceRNAs mediated by multiple moderate miRNA regulators cannot be detected. Therefore, we utilised an extension of scor, the multiple sensitivity correlation (mscor) method, which has been implemented in the SPONGE (sparse partial correlation on gene expression) R/Bioconductor package [20]. The derived formula with which to calculate mscor is given in Equation (2):

(2) $$mscor\left(g_{1},g_{2},M\right)=cor\left(g_{1},g_{2}\right)-pcor(g_{1},g_{2}|M)\;$$

where *M* = *m*_1_,*m*_2_…,*m_i_* and *i* is the number of shared miRNAs between *g*_1_ and *g*_2_ genes. The *cor*() term defines the Pearson correlation between *g*_1_ and *g*_2_ genes expression profiles, and *pcor*() is the partial correlation that estimates how two variables are correlated when they are controlled by additional variables. Furthermore, the SPONGE method defines a null distribution, which allows for the estimation of an empirical *p*-value for mscor.

We filtered ceRNA interactions returned by three user-defined significant thresholds, (i) in the hypergeometric test, FDR-adjusted *p*-value < 0.05, (ii) the Pearson correlation coefficient between ceRNA pairs > 0.4, and (iii) the adjusted *p*-value of mscor in the SPONGE method < 0.05. The resulting lncRNA–mRNA–miRNA and pseudogene–mRNA–miRNA combinations in each HD cancer-specific ceRNA network were integrated into a single variable. The format of the derived categorical variable is “<lncRNA/pseudogene gene ensemble ID>_<gene ensemble ID>_<miRNA name>”. After that, we constructed a one-way table to identify shared lncRNA/pseudogene–mRNA–miRNA associations across all five HD cancer types. Suppose a one-way frequency equals 5 for a given lncRNA/pseudogene–mRNA–miRNA pair. In that case, a ceRNA association is classified as “the shared ceRNA network of HD cancers”. We used the Cytoscape software to visualise the shared ceRNA network of HD cancers [24]. Moreover, Venn diagram representations were utilised to visualise the counts of shared/individual RNAs across HD cancer types. Two downstream analyses were conducted for genes and miRNAs included in the shared ceRNA network of HD cancers.

### 2.6. Functional Enrichment Analysis

The functional enrichment analysis was performed for genes in the shared ceRNA network of HD cancers. The Gene Ontology (GO) and Kyoto Encyclopedia of Genes and Genomes (KEGG) functional enrichment analyses were conducted using the R/Bioconductor clusterProfiler R package [25].

### 2.7. Survival Analysis

We performed survival analysis using the Kaplan–Meier (K–M) survival curves, implemented in the survival R package [26] to explore the role of genes in the shared ceRNA network. For each gene/miRNA, the tumour samples were divided into two groups (low-expressed and high-expressed) according to the median gene/miRNA expression value. The logrank test (Mantel–Haenszel test) was used as the statistical method for the Kaplan–Meier curves. The logrank test statistic has a chi-square ($\chi^{2})$ distribution with one degree of freedom. Therefore, significant genes and miRNAs were chosen under the $\chi^{2}\;$test statistic *p*-value < 0.05. We used miRCancer [27], a literature-curated database for miRNA experimental studies in cancers, to check descriptions of prognostic miRNAs.

## 3. Results

After the quality-control process, we retrieved 495/52 (cases/controls), 1091/113, 456/41, 166/10, and 543/35 samples from PRAD, BRCA, COAD, READ, and UCEC. In the differential expression analysis, we used 15509, 15244, 14771, 14866, and 15197 genes from PRAD, BRCA, COAD, READ, and UCEC after removing those that were low-expressed.

### 3.1. Differential Expression Analysis Results

The differential expression analysis between tumours and adjacent normal samples was conducted using the limma R package. The count of differentially expressed (up/down) lncRNAs, pseudogenes, mRNAs, and miRNAs are given in Table 1.

### 3.2. Shared Competing Endogenous RNA Networks across Hormone-Dependent Cancers

First, we identified significant lncRNA–mRNA–miRNA and pseudogene–mRNA–miRNA networks for each HD cancer. The number of lncRNAs/pseudogenes, mRNAs, and miRNAs in ceRNA networks of individual HD cancers are reported in Supplementary Materials Table S1. We used both the GDCRNATools and SPONGE R packages for ceRNA network analysis. Table 2 contains all shared ceRNA associations found from the GDCRNATools approach (steps i and ii). In Table 2, common ceRNAs from both GDCRNATools and SPONGE partial correlation analyses (step iii) are labelled by an asterisk (*).

**Figure 2:**
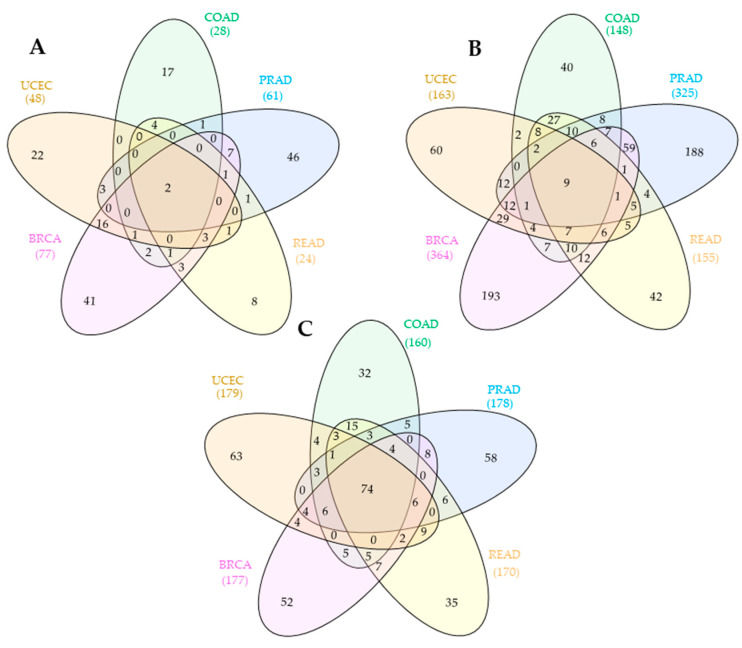
Venn diagram representations for the number of lncRNAs (**A**), mRNAs (**B**), and microRNAs (**C**) involved in individual/shared HD cancer competing endogenous RNA network(s).

According to Table 2, integrative analysis of GDCRNATools and SPONGE packages (GDCRNATools + SPONGE) resulted in two lncRNAs, nine mRNAs, and seventy-four miRNAs. Figure 2 represents Venn diagrams for the number of shared/individual lncRNAs, mRNAs, and miRNAs across/within HD cancers that contributed to the construction of the shared ceRNA network of HD cancers.

**Figure 3:**
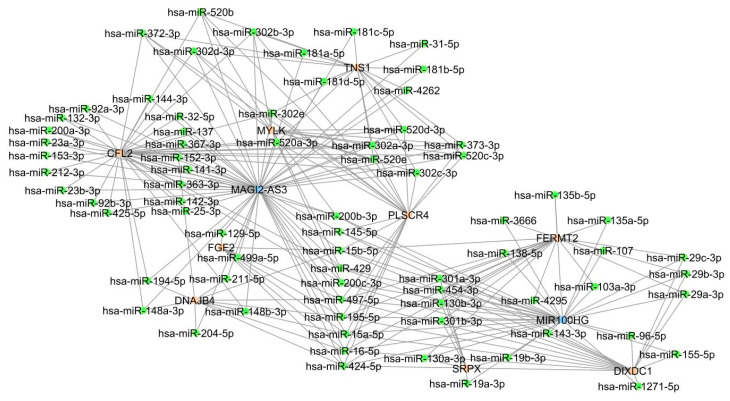
The shared competing endogenous RNA network across five hormone-dependent (HD) cancers, breast, prostate, colon, rectal, and endometrial, was constructed by the Cytoscape tool [24]. The blue-, orange-, and green-coloured circles represent long non-coding RNAs, mRNAs, and microRNAs, respectively.

According to Table 2 and Figure 2, only a limited number of lncRNAs and mRNAs are shared across five HD cancer ceRNA networks. Figure 3 illustrates a graphical representation of the shared ceRNA networks of HD cancers found in our study, which was prepared using the Cytoscape software [24].

According to Figure 3, the majority of miRNAs bind with the MAGI2-AS3-associated ceRNA network. We conducted two downstream analyses for genes and miRNAs in the shared ceRNA network of HD cancers which are represented in Figure 3.

### 3.3. Functional Enrichment Analysis

**Figure 4:**
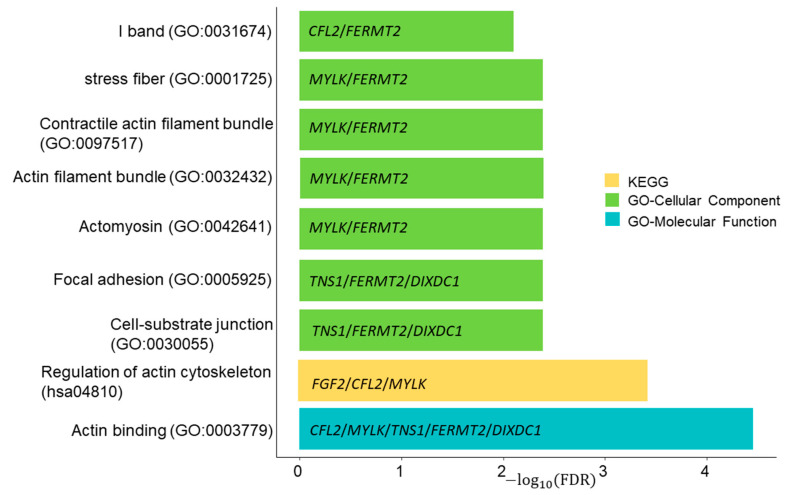
Functional enrichment analysis of nine genes in the shared competing endogenous RNA network of hormone-dependent (HD) cancers. There were seven, one, and one statistically significant component(s) in the GO CC, GO MF, and KEGG pathways, respectively. Six out of nine genes, *CFL2*, *MYLK*, *TNS1*, *FERMT2*, *DIXDC1*, and *FGF2* are associated with actin-related pathways.

Functional enrichment analysis was performed on the nine mRNAs obtained from the shared ceRNA network of HD cancers. The GO cellular components (CC) of enrichment were mainly I band, stress fibre, contractile actin filament bundle, actin filament bundle, actomyosin, focal adhesion, and cell–substrate junction. Five out of nine genes (*CFL2*, *MYLK*, *TNS1*, *FERMT2*, and *DIXDC1*) were enriched in the actin-binding component in the GO molecular functions (MF) pathway. KEGG pathway analysis showed that 3 out of 9 mRNAs (*FGF2*, *CFL2*, and *MYLK*) are involved in the regulation of the actin cytoskeleton pathway. Results of the enrichment analysis are illustrated in Figure 4.

### 3.4. Survival Analysis

**Figure 5:**
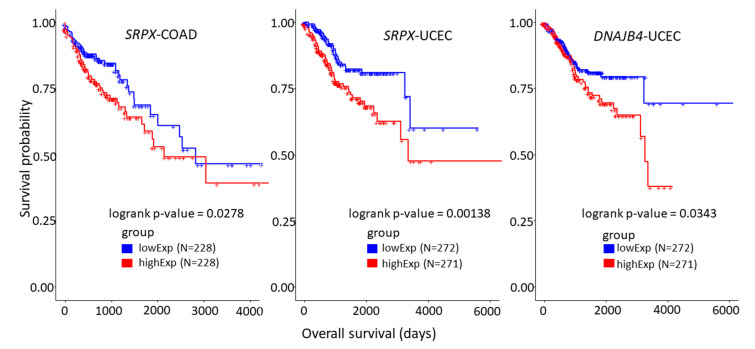
Kaplan–Meier (K–M) survival analysis plots for prognostic mRNAs in the shared ceRNA network of hormone-dependent (HD) cancers. The low-expressed SRPX gene exhibits prognostic ability in both colon (**left**) and endometrial (**centre**) cancer. The low-expressed DNAJB4 shows better survival in endometrial cancer (**right**).

We performed Kaplan–Meier (K–M) survival analysis for genes and miRNAs in the shared ceRNA network of HD cancers. The gene and miRNA lists were applied to individual HD cancer survival analysis. We filtered out genes and miRNAs that were significant from at least one survival analysis. We found that two genes and forty-one miRNAs in the shared ceRNA network are significant in at least one HD cancer. Two mRNAs out of nine, *SRPX* and *DNAJB4*, were significant in COAD and UCEC survival analyses. Figure 5 illustrates K–M curves for the two prognostic genes in COAD and UCEC.

We performed a K–M survival analysis for the list of miRNAs (seventy-four) in the shared ceRNA network of HD cancers. We selected miRNAs that are significant in at least one HD cancer survival analysis. We found that forty-one out of seventy-four miRNAs exhibit predictive ability in at least one HD cancer. Table 3 shows the significant miRNAs with hazard ratios and logrank test *p*-values. We used the miRCancer web-based tool [27] to explore miRNA–HD cancer associations of listed significant miRNAs from the survival analysis. In Table 3, the superscripted 1, 2, 3, 4, and 5 implies that the given miRNA has been experimentally validated in prostate, breast, colon, rectal, and endometrial cancer, respectively. Each miRNA has been labelled as “low” or “high” to indicate its expression level in cancer survival.

As stated in Table 3, hsa-miR-301b-3p acts as a prognostic candidate in BRCA and UCEC, whereas hsa-miR-497-5p is significant in both COAD and UCEC survival analyses. According to the results, eleven, seven, one, two, and twenty miRNAs were obtained from the BRCA, COAD, READ, PRAD, and UCEC survival analyses, respectively.

## 4. Discussion

Previous genome-wide and transcriptome-wide analyses have reported the existence of a shared genetic aetiology of HD cancers [28]. As ceRNAs have a critical role in gene and molecular pathways, identifying a shared ceRNA network of HD cancers will contribute to understanding the shared genetic aetiology of HD cancers. Here, we investigated the availability of a shared ceRNA network of five common HD cancers. Previous HD-cancer-related ceRNA analyses have focused on lncRNA-associated networks [6,7,8,9,10]. We extended the scope of ceRNA research to include pseudogene-associated cross-HD cancer ceRNA networks.

We utilised two ceRNA analysis R packages, GDCRNATools and SPONGE, to improve the predictive power of ceRNA analyses [19,20]. Prior HD-cancer-associated ceRNA analyses have used the sensitivity correlation method defined in the GDCRNATools. The sensitivity correlation cannot account for the presence of multiple miRNAs for a given ceRNA pair. To address this limitation, we used the sparse partial correlation method implemented in the SPONGE R/Bioconductor package. We aggregated HD-cancer-specific lncRNA/pseudogene–miRNA–mRNA associations (significant from both GDCRNATools and SPONGE) to evaluate the shared lncRNA/pseudogene–miRNA–mRNA triplets across five HD cancer types. We identified two lncRNAs, nine mRNAs, and seventy-four miRNAs common across lncRNA–mRNA–miRNA networks in HD cancers. None of the pseudogene-related shared ceRNA associations selected from GDCRNATools were significant from the SPONGE method. Previous cancer studies have extensively described two lncRNAs in the shared ceRNA network, MAGI2-AS3, and MIR100HG [29,30,31,32,33,34,35]. Du et al. [30] have shown that MAGI2-AS3 upregulation inhibits BRCA metastatic progression by decreasing miR-374a and enhancing *PTEN* expression. Ren et al. [31] have revealed that MAGI2-AS3 promotes colorectal cancer progression by regulating the miR-3163-*TMEM106B* axis. Moreover, the MAGI2-AS3 promoter was hypermethylated in several cancers such as COAD, READ, and UCEC [32].

The lncRNA MIR100HG, the host gene for miR-100, let-7a-2, and miR-125b cluster, has been previously reported to have a role in gastric cancer, colorectal cancer, and BRCA [33,34,35]. Li et al. [34] demonstrated that MIR100HG overexpression causes colorectal cancer progression and is a poor prognosis in colorectal cancer patients. It also promotes triple-negative BRCA cells’ migration, invasion, and proliferation by sponging the miR-5590-3p-*OTX1* axis [35]. Our study reveals the ceRNA role of MIR100HG in PRAD and UCEC as well as that of MAGI2-AS3 in PRAD for the first time. Wet-lab experiments are required to understand the molecular mechanism of MAGI2-AS3 and MIR100HG in these cancers.

We found nine mRNAs in the shared ceRNA network of HD cancers in which six and three mRNAs were paired with MAGI2-AS3 and MIR100HG, respectively. Eight out of nine mRNA–lncRNA axes were identified for the first time in cancer-related ceRNAs. These ceRNA pairs are likely to be involved in cancer pathways as they were significant across five cancer types. The MAGI2-AS3/miR-31-5p/*TNS1* axis identified in our study has been shown to regulate migration and invasion ability in bladder cancer cell lines [36].

We conducted two downstream analyses, a survival analysis and a functional enrichment analysis, to identify important mRNAs and miRNAs in the shared ceRNA network. Two out of nine mRNAs, *SRPX* (UCEC and COAD) and *DNAJB4* (UCEC), were found as prognostic markers in at least one HD cancer from the survival analysis. The *SRPX* gene acts as a tumour-suppressor gene, which is down-regulated in several malignancies, including PRAD, COAD, READ, and neuroendocrine (cells that release hormones into the blood in response to stimulation of the nervous system) cancers [37]. All these malignancies are biologically related to hormones. Therefore, the role of the *SRPX* gene in hormone-related cancers should be further investigated. Currently, *SRPX* is being examined as a potential cancer drug under patent number US 9,290,744 B2 [38].

The *DNAJB4* gene (also known as *HLJ1*) belongs to the DNJ family heat shock proteins (HSPs) and is regarded as a tumour-suppressor in COAD, BRCA, lung, and gastric cancer [39]. HSPs have been reported as biomarkers and potential drug targets of cancers for decades. A recent integrative analysis of multi-omics data uncovered the distinct impact of several HSP (including *DNAJB4*) members on BRCA progression [40]. Our study discovered that *DNAJB4* could act as a prognostic marker in UCEC. Moreover, GDCRNATools analysis (only the hypergeometric test and Pearson correlation analysis) identified that *DNAJB4* can be paired with all three lncRNAs in the shared network, MBNL1-AS1, MAGI2-AS3, and MIR100HG. Therefore, in vivo/vitro experiments are required to evaluate its tumour-suppressive/oncogenic role in HD cancers.

We conducted a separate survival analysis for miRNAs in the shared ceRNA network. Interestingly, our miRNA survival analysis revealed that ~55% (41/74) of miRNAs in the shared ceRNA network of HD cancers are associated with disease survival in at least one HD cancer type. These forty-one miRNAs have been experimentally validated for their functional role in at least three HD cancers (out of five types of interest), providing confidence to our computational results [27]. Among these forty-one miRNAs, twenty-eight have been differentially expressed in HD cancers. We found multiple prognostic miRNAs from the same miRNA family, two from miR-181 (in COAD and BRCA), three from miR-29 (in BRCA and PRAD), three from miR-301 (in BRCA and UCEC), five from miR-302 (in UCEC), and four from miR-520 (in UCEC). The four members of the miR-520 family that were prognostic in UCEC are required to be determined through experiments. We found both miR-302 and miR-367 as prognostic markers from the shared ceRNA network. The miR-302/367 cluster has been previously identified in PRAD-, BRCA-, COAD-, READ-, and UCEC-associated pathways, supporting our findings [41].

According to functional enrichment analysis, six out of nine mRNAs were associated with actin-related GO and KEGG pathways. The actin dynamics and actin-specific molecular signalling have shown potential clinical significance on non-genomic steroid hormone actions on tumour cells [42]. All these facts supported by the literature have improved the significance of our study’s shared ceRNA network of HD cancers.

A limitation of this study is that we selected both experimentally validated and predicted miRNA–target interactions only from two databases, miRcode and starBase, to include a substantial set of miRNA–mRNA/lncRNA/pseudogene interactions. We did not include circular RNAs (circRNAs) for the ceRNA network analysis as their expression levels are not available in TCGA. Nevertheless, our findings have important biological implications for HD cancers.

Herein, we identified a shared ceRNA network that can be facilitated to understand the shared genetic aetiology of HD cancers. The shared ceRNA network consists of two lncRNAs, nine mRNAs, and seventy-four miRNAs that have shown links with individual HD malignancies from the literature. Our study lays the groundwork for future research on understanding the role of these mRNAs, miRNAs, and lncRNAs in the shared genetic susceptibility of HD cancers. Future directions could lead to a supervised machine learning approach to understand molecular effects on ceRNA networks of HD cancers.

## 5. Conclusions

We conducted the first extensive computational study that compares ceRNA networks (both lncRNA and pseudogene) in a group of related cancers, HD cancers. The shared ceRNA network comprises two lncRNAs, nine mRNAs, and seventy-four miRNAs, and some of them were described for the first time in certain HD cancers. A global view of the functional ceRNA networks of large sample sets encompassing multiple tumour types may help identify potential unexpected targets that apply to a cancer subset, such as HD cancers. Moreover, identifying novel risk-associated lncRNAs, pseudogenes, miRNAs, and mRNAs across a group of related cancers will significantly contribute to understanding their shared disease biology. Further experimental investigations should be conducted to understand the tumour-suppressive/oncogenic/cancer-driven role of identified ceRNAs in HD cancers.

## Figures and Tables

**Table 1:** Count of differentially expressed (up/down) lncRNAs, pseudogenes, mRNAs, and miRNAs in each hormone-dependent (HD) cancer (corrected for multiple testing).

Cancer	lncRNA		Pseudogene		mRNA		miRNA	
	Up	Down	Up	Down	Up	Down	Up	Down
BRCA	61	106	17	28	1125	1642	71	87
COAD	137	72	44	31	1200	1778	186	153
PRAD	139	49	28	18	434	1079	34	27
READ	181	53	52	18	1169	1790	165	114
UCEC	116	137	43	43	1584	2000	142	103

**Table 2:** Shared lncRNA and pseudogene-associated ceRNA associations among hormone-dependent (HD) cancers.

lncRNA/Pseudogene	mRNA	List of MicroRNAs Associated with Each lncRNA/Pseudogene–mRNA Pair
MBNL1-AS1 (lncRNA)	DnaJ heat shock protein family (Hsp40) member B4 (*DNAJB4*)	hsa-miR-15a-5p, 16-5p, 15b-5p, 195-5p, 424-5p, 497-5p
MAGI2-AS3* (lncRNA)	*DNAJB4* *	hsa-miR-148a-3p, 152-3p, 148b-3p, 15a-5p, 16-5p, 15b-5p, 195-5p, 424-5p, 497-5p, 194-5p, 204-5p, 211-5p
	Fibroblast growth factor-2 * (*FGF-2* *)	hsa-miR-15a-5p, 16-5p, 15b-5p, 195-5p, 424-5p, 497-5p, 129-5p, 499a-5p
	Myosin light-chain kinase * (*MYLK* *)	hsa-miR-302a-3p, 302b-3p, 302c-3p, 302d-3p, 372-3p, 373-3p, 520e, 520a-3p, 520b, 520c-3p, 520d-3p, 302e, 200b-3p, 200c-3p, 429
	Junctophilin-2 (*JPH2*)	hsa-miR-25-3p, 32-5p, 92a-3p, 363-3p, 367-3p, 92b-3p
	Cofilin-2 * (*CFL2* *)	hsa-miR-212-3p, 132-3p, 302a-3p, 302b-3p, 302c-3p, 302d-3p, 372-3p, 373-3p, 520e, 520a-3p, 520b, 520c-3p, 520d-3p, 302e, 137, 141-3p, 200a-3p, 142-3p, 144-3p, 148a-3p, 152-3p, 148b-3p, 153-3p, 194-5p, 200b-3p, 200c-3p, 429, 23a-3p, 23b-3p, 25-3p, 32-5p, 92a-3p, 363-3p, 367-3p, 92b-3p, 425-5p
	Phospholipid scramblase 4 * (*PLSCR4* *)	hsa-miR-302a-3p, 302b-3p, 302c-3p, 302d-3p, 372-3p, 373-3p, 520e, 520a-3p, 520b, 520c-3p, 520d-3p, 302e, 145-5p, 15a-5p, 16-5p, 15b-5p, 195-5p, 424-5p, 497-5p
	Endothelin receptor type B (*EDNRB*)	hsa-miR-302a-3p, 302b-3p, 302c-3p, 302d-3p, 372-3p, 373-3p, 520e, 520a-3p, 520b, 520c-3p, 520d-3p, 302e
	Tensin 1 * (*TNS1* *)	hsa-miR-302a-3p, 302b-3p, 302c-3p, 302d-3p, 372-3p, 373-3p, 520e, 520a-3p, 520b, 520c-3p, 520d-3p, 302e, 181a-5p, 181b-5p, 181c-5p, 181d-5p, 4262, 31-5p
MIR100HG* (lncRNA)	FERM-domain-containing kindlin-2 * (*FERMT2* *)	hsa-miR-130a-3p, 301a-3p, 130b-3p, 454-3p, 301b-3p, 4295, 3666, 135a-5p, 135b-5p, 138-5p, 15a-5p, 16-5p, 15b-5p, 195-5p, 424-5p, 497-5p, 29a-3p, 29b-3p, 29c-3p, 103a-3p, 107
	DIX-domain-containing 1 * (*DIXDC1* *)	hsa-miR-96-5p, 1271-5p, 143-3p, 145-5p, 155-5p, 15a-5p, 16-5p, 15b-5p, 195-5p, 424-5p, 497-5p, 200b-3p, 200c-3p, 429, 29a-3p, 29b-3p, 29c-3p
	R-spondin 3 (*RSPO3*)	hsa-miR-15a-5p, 16-5p, 15b-5p, 195-5p, 424-5p, 497-5p, 103a-3p, 107
	*DNAJB4*	hsa-miR-148a-3p, 152-3p, 148b-3p, 15a-5p, 16-5p, 15b-5p, 195-5p, 424-5p, 497-5p, 204-5p, 211-5p, 103a-3p, 107
	*FGF2*	hsa-miR-15a-5p, 16-5p, 15b-5p, 195-5p, 424-5p, 497-5p, 103a-3p, 107, 129-5p
	Sushi repeat-containing protein X-linked * (*SRPX* *)	hsa-miR-130a-3p, 301a-3p, 130b-3p, 454-3p, 301b-3p, 19a-3p, 19b-3p
	*JPH2*	hsa-miR-25-3p, 32-5p, 92a-3p, 363-3p, 367-3p, 92b-3p
MEIS3P1 (pseudogene)	*TNS1*	hsa-miR-138-5p, 138-1-5p, 145-5p, 204-5p, 204-3p, 211-5p, 219a-5p, 508-5p, 508-3p, 4782-3p, 23a-5p, 23b-5p, 34a-5p, 34b-5p, 449a, 449c-5p
	KN motif and ankyrin repeat domains 2 (*KANK2*)	hsa-miR-138-5p, 138-1-5p, 145-5p, 204-5p, 204-3p, 211-5p, 219a-5p, 508-5p, 508-3p, 4782-3p, 34a-5p, 34b-5p, 449a, 449c-5p
TUBAP5 (pseudogene)	MYB proto-oncogene-like 2 (*MYBL2*)	hsa-miR-130a-3p, 301a-5p, 301b-5p, 301b-3p, 454-5p, 721, 4295, 3666, 7-5p, 7-1-3p, 148a-3p, 152-5p, 15a-5p, 16-5p, 16-1-3p, 195-5p, 322, 424-5p, 497-3p, 1907, 214-5p, 761, 3619-5p, 22-5p, 22-3p, 122-5p, 122-3p, 1352, 24-3p, 24-1-5p, 24-2-5p, 29a-3p, 103a-3p, 107, 107ab, 124-5p, 124-3p, 506-5p, 338-5p, 338-3p

In Table 2, common ceRNAs from both methods, GDCRNATools and SPONGE partial correlation analyses (step iii), are labelled by an asterisk (*).

**Table 3:** Logrank test results (survival analysis) for miRNAs in the shared ceRNA network of hormone-dependent (HD) cancers.

Cancer	miRNA (High/Low Expression Levels Associated with Survival)	Hazard Ratio	*p*-Value	Cancer	miRNA (High/Low Expression Levels Associated with Survival)	Hazard Ratio	*p*-Value
BRCA	hsa-miR-16-5p (high) ^1,2,3,4^	0.672	0.0136	UCEC	hsa-miR-142-3p (high) ^1,2,3,4,5^	0.5634	0.0078
BRCA	hsa-miR-181c-5p (high) ^1,2,3,4,5^	0.6578	0.0114	UCEC	hsa-miR-148a-3p (high) ^1,2,3,4,5^	0.55	0.0055
BRCA	hsa-miR-195-5p (high) ^1,2,3,4,5^	0.6859	0.0212	UCEC	hsa-miR-152-3p (low) ^1,2,3,4,5^	1.6863	0.0156
BRCA	hsa-miR-200c-3p (high) ^1,2,3,4,5^	0.7097	0.04	UCEC	hsa-miR-212-3p (low) ^1,2,3,4^	1.7536	0.0096
BRCA	hsa-miR-204-5p (high) ^1,2,3,4,5^	0.6294	0.0052	UCEC	hsa-miR-25-3p (low) ^2,3,4,5^	1.5573	0.0365
BRCA	hsa-miR-29a-3p (high) ^1,2,3,4,5^	0.7168	0.0429	UCEC	hsa-miR-301a-3p (low) ^2,3,4,5^	1.8982	0.0032
BRCA	hsa-miR-29c-3p (high) ^1,3,4,5^	0.6313	0.0061	UCEC	hsa-miR-301b-3p (low) ^1,2,5^	1.6064	0.0277
BRCA	hsa-miR-301b-3p (low) ^1,2,5^	1.3884	0.0478	UCEC	hsa-miR-302a-3p (high) ^1,2,3,4,5^	0.5663	0.0071
BRCA	hsa-miR-31-5p (high) ^1,2,3,4^	0.5542	0.0003	UCEC	hsa-miR-302b-3p (high) ^1,2,3,4,5^	0.5608	0.0061
BRCA	hsa-miR-363-3p (high) ^2,3,4,5^	0.6961	0.0279	UCEC	hsa-miR-302c-3p (high) ^1,2,3,4,5^	0.5498	0.0049
BRCA	hsa-miR-372-3p (low) ^1,2,3,4^	1.409	0.0392	UCEC	hsa-miR-302d-3p (high) ^1,2,3,4,5^	0.5531	0.0053
COAD	hsa-miR-1271-5p (low) ^1,2,3,4,5^	1.6083	0.0166	UCEC	hsa-miR-302e (high) ^1,2,3,4,5^	0.4897	0.0008
COAD	hsa-miR-130a-3p (low) ^1,2,3,4,5^	1.8346	0.0021	UCEC	hsa-miR-367-3p (high) ^1,2,3,4,5^	0.5204	0.0021
COAD	hsa-miR-145-5p (low) ^1,2,3,4^	1.5823	0.0214	UCEC	hsa-miR-425-5p (low) ^1,2,3,4^	1.6045	0.0301
COAD	hsa-miR-181b-5p (low) ^1,2,5^	1.5294	0.0326	UCEC	hsa-miR-4262 (high) ^1,2,3,4,5^	0.4897	0.0008
COAD	hsa-miR-32-5p (low) ^1,2,3,4,5^	1.5932	0.0213	UCEC	hsa-miR-497-5p (high) ^1,2,3,4^	0.5285	0.0037
COAD	hsa-miR-497-5p (low) ^1,2,3,4^	1.5895	0.0206	UCEC	hsa-miR-520b (high) ^1,2,3,4^	0.5896	0.0129
COAD	hsa-miR-96-5p (low) ^1,2,3,4^	1.4888	0.0474	UCEC	hsa-miR-520c-3p (high) ^1,2,3,4^	0.5791	0.0099
PRAD	hsa-miR-19a-3p (low) ^1,2,3,4^	6.9585	0.026	UCEC	hsa-miR-520d-3p (high) ^2,3,4^	0.5043	0.0012
PRAD	hsa-miR-29b-3p (high) ^1,2,3,4^	0.2343	0.0434	UCEC	hsa-miR-520e (high) ^2,3,4^	0.626	0.0273
READ	hsa-miR-155-5p (high) ^1,2,3,4,5^	0.4544	0.0426				

The superscripted ^1,2,3,4^ and ^5^ implies that the given miRNA has been experimentally validated in prostate, breast, colon, rectal, and endometrial cancer, respectively. Each miRNA has been labelled as “low” or “high” to indicate its expression level in cancer survival.

## Data Availability

Publicly available TCGA RNA-seq and miRNA-seq expression data were downloaded through the GDC Data Portal (https://portal.gdc.cancer.gov/repository, accessed on 18 May 2020). All statistical analyses and graph preparations were performed using the R statistical software, freely available at https://cran.r-project.org/ (accessed on 20 May 2019). The ceRNA network graphs were drawn using the Cytoscape software, freely available at https://cytoscape.org/download.html (accessed on 22 May 2020).

## Supplementary Materials

The following are available online at https://www.mdpi.com/article/10.3390/biology10101014/s1, Table S1: Number of statistically significant long non-coding RNA/pseudogene–mRNA–microRNA triplets in each cancer type (the ceRNA associations from GDCRNATools and both GDCRNATools and SPONGE—sparse partial correlation on gene expression—have been tabulated separately).
