# Supplementary material for: Identifying Complex lncRNA/Pseudogene–miRNA–mRNA Crosstalk in Hormone-Dependent Cancers
Source: Biology (Basel). 2021 Oct 9;10(10):1014. doi: 10.3390/biology10101014 (PMC8533463; doi:10.3390/biology10101014)
Supplement: Supplementary file 1 [file biology-10-01014-s001.zip › biology-1401896-supplementary.pdf]

Table S1. Number of statistically significant long non-coding RNA/pseudogene-mRNA-microRNA triplets in each cancer type (the ceRNA associations from GDCRNATools and both GDCRNATools and SPONGE-Sparse Partial correlation ON Gene Expression have been tabulated separately).

**Cancer**	**Number of lncRNAs, mRNAs, and miRNAs in ceRNA network**		**Number of pseudogenes, mRNAs, and miRNAs in ceRNA network**	
	**GDCRNATools**	**GDCRNATools+SPONGE**	**GDCRNATools**	**GDCRNATools+SPONGE**
**BRCA**	93, 432, 178	77, 364, 177	33, 764, 207	27, 571, 206
**COAD**	50,219, 168	28, 148, 160	57, 1044, 208	40, 521, 204
**PRAD**	86, 354, 178	61, 325, 178	42, 1339, 208	30, 1183, 206
**READ**	89, 353, 176	24, 155, 170	60, 1206, 207	28, 344, 203
**UCEC**	99, 359, 182	48, 163, 179	60, 654, 209	24, 207, 206
**Shared**	3, 12, 74	2, 9, 74	2, 3, 57	None

*lncRNA, long non-coding ribonucleic acid; mRNA, messenger RNA; miRNA, microRNA; SPONGE, Sparse Partial correlation ON Gene Expression; BRCA, breast cancer; COAD, colon cancer; READ, rectal cancer; UCEC, endometrial cancer, Shared, common among BRCA, PRDA, (COAD/READ), and UCEC. GDCRNATools+SPONGE implies statistically significant ceRNA associations from both GDCRNATools and SPONGE.
